# Molecular characterization of *Sarcocystis* spp. in intestines of coyotes and foxes from Pennsylvania, USA

**DOI:** 10.1186/s13071-026-07515-w

**Published:** 2026-06-18

**Authors:** Aditya Gupta, Kyle Van Why, Justin D. Brown, Benjamin. M. Rosenthal, Jitender P. Dubey

**Affiliations:** 1 United States Department of Agriculture, Agricultural Research Service, Animal Parasitic Diseases Laboratory, Beltsville Agricultural Research Center, Beltsville, MD 20705-2350 USA; 2 United States Department of Agriculture Wildlife Services, PO Box 60827, Harrisburg, PA 17106 USA; 3 Department of Veterinary and Biomedical Sciences, Pennsylvania State University, University Park, PA 16802 USA

**Keywords:** Coyotes, Foxes, Pennsylvania, Sporocyst, Genotyping, Ileum, *Sarcocystis*

## Abstract

**Background:**

Wild canids serve as definitive hosts for numerous *Sarcocystis* species infecting livestock and wildlife. However, species-level identification in free-ranging carnivores remains difficult, impeded by the morphological resemblance among the sporocysts of various parasite species.

**Methods:**

Intestinal mucosal scrapings from 40 wild canids: red fox (*Vulpes vulpes*, n = 8), gray fox (*Urocyon cinereoargenteus*, n = 6) and coyote (*Canis latrans*, n = 26) in Pennsylvania, USA, sampled in 2024, were tested for *Sarcocystis* sporocysts.

**Results:**

Sporocysts were detected microscopically in intestinal homogenates digested in Chlorox in 3/6 (50%) gray foxes, 3/8 (37.5%) red foxes and 18/26 (69.2%) coyotes. PCR amplification was successful on 8/18 (44.4%) coyotes, 0/6 Gy foxes and 2/3 (66.6%) red foxes. Multi-locus genotyping was performed for the *18S* rRNA, *28S* rRNA, *COI* and *ITS1* genetic markers, supporting identification of multiple species of *Sarcocystis*, including *Sarcocystis albifronsi*-like, *S. cruzi*, *S. gjerdei*-like, *S. cristata*/*S. wenzeli*-like and an additional undescribed, ungulate-associated *Sarcocystis* spp.

**Conclusions:**

Phylogenetic analysis confirmed clustering with reference sequences with strong support. This study demonstrates that wild foxes and coyotes serve as definitive hosts for multiple species of *Sarcocystis*, including *Sarcocystis cruzi,* which uses cattle as intermediate hosts and imposes significant economic burdens on cattle production. Moreover, these findings demonstrate that wild canids harbor diverse *Sarcocystis* spp., supporting their role in environmental dissemination and potential transmission at the wildlife–livestock interface.

**Graphic abstract:**

**Figure:**
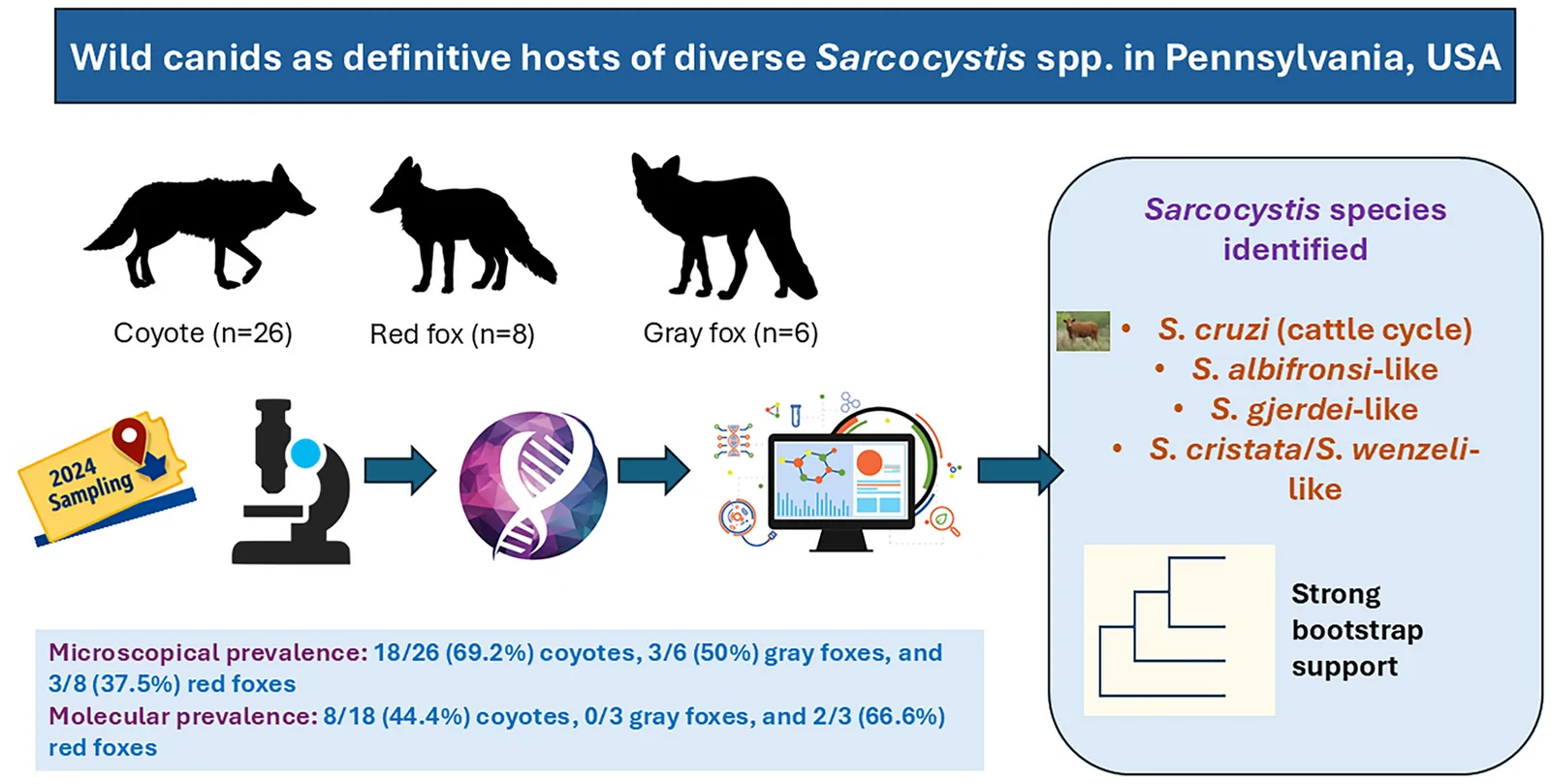

## Background

Species of *Sarcocystis* (Apicomplexa: Sarcocystidae) employ obligatory two-host life cycles involving sexual reproduction in a definitive host (DH) and tissue cyst formation in an intermediate host (IH) [1]. Canids serve as DH for numerous *Sarcocystis* species infecting domestic livestock and wild ungulates. Because sporocysts are morphologically similar across species, assigning them to species generally requires molecular characterization and microscopic examination of intestinal mucosal scrapings [2–9]. Molecular investigations in foxes and coyotes have confirmed their competence as DH for several species of *Sarcocystis*, confirmed by sporocyst shedding after experimental feeding of infected tissues [10–16].

Among these, *Sarcocystis cruzi* is particularly important because it infects cattle worldwide and has a well-established canid–bovine life cycle and is economically important [17, 18]. Experimental studies showed that dogs and coyotes excrete *S. cruzi* sporocysts after ingesting infected beef, and subsequent infections in calves demonstrated vascular endothelial stages [19, 20]. Experimentally, red foxes can also act as DH for *S. cruzi*, *S. capracanis* of goats and *S. tenella* of sheep [17] but nothing is known of the role of foxes in the natural epidemiology of livestock *Sarcocystis* species. Recently wolves were found to be DH for many *Sarcocystis* species including *S. cruzi* in Minnesota, USA [3].

Pennsylvania supports abundant fox population, significant cattle production, and expanding coyote distributions, creating ecological conditions favorable for predator–prey transmission cycles. Despite this, molecular data on *Sarcocystis* spp. circulating in wild canids in the northeastern United States remain limited. Therefore, our objective was to molecularly characterize *Sarcocystis* spp. detected in intestinal homogenates of foxes and coyotes in Pennsylvania using various molecular markers and to evaluate the potential epidemiological significance of the parasite species thereby identified.

## Methods

### Sample

The last 15–30 cm segment of ileum was collected from red foxes (*Vulpes vulpes*, n = 8), gray foxes (*Urocyon cinereoargenteus*, n = 6) and coyotes (*Canis latrans*, n = 26) in 2024 in Pennsylvania, USA. Animals included in this study were collected from hunter harvested animals and sampled postmortem by personnel of the U.S. Department of Agriculture Wildlife Services (under Pennsylvania Game Commission Special Use Permits 39,964 and 31,360). Segments of ileum were removed and tied off on either end. Ileum samples were kept on ice packs and shipped to the Animal Parasitic Diseases Laboratory (APDL), Beltsville, Maryland within 72 h of harvest. Samples were received on 7th February at APDL. In addition, the following information for each animal was recorded through examination of the carcass and interview of the hunter: species, sex, age, date of harvest, county, and approximate location (closest town or township) of harvest within Pennsylvania (detailed information provided in Table 1 of Dubey et al. [21]).

**Table 1:** Prevalence of *Sarcocystis* sporocyst in intestines of coyotes and foxes in Pennsylvania, USA

Animal	Lab ID	Age and sex	County	Results		Molecular markers studied		
				Fecal floatation/microscopy	Molecular	*18S rRNA*	*ITS1*	*COI*
Coyotes	C-PA23-1	Juvenile, F	Centre	Pos	Neg	**–**	**–**	**–**
	C-PA23-2	Adult, Male	Centre	Pos	Neg	**–**	**–**	**–**
	C-PA23-3	Adult, Male	Centre	Pos	Neg	**–**	**–**	**–**
	USDA-PA2	Adult, F	Dauphin	Pos	Neg	**–**	**–**	**–**
	JP-5-2	Adult, M	Wyoming	Pos	Neg	**–**	**–**	**–**
	JP-5-6	Adult, M	Lackawanna	Pos	Neg	**–**	**–**	**–**
	JP-5-7	Adult, F	Lackawanna	Neg	Neg	**–**	**–**	**–**
	JP-5-12	Adult, M	Wyoming	Pos	Neg	*S. cruzi*	NA	*S.* sp.
	JP-5-17	Adult, F	Bradford	Pos	Pos	NA	NA	NA
	JP-5-18	Adult, M	Wayne	Pos	Neg	**–**	**–**	**–**
	JP-5-20	Adult, M	Wayne	Neg	Neg	**–**	**–**	**–**
	JP-5-35	Adult, F	Pike	Pos	Pos	NA	NA	*S.* sp.
	JP-5-39	Juvenile, F	Luzerne	Pos	Pos	NA	NA	*S.* sp
	JP-5-40	Adult, M	Lackawanna	Neg	Neg	**–**	**–**	**–**
	JP-5-45	Juvenile, M	Susquehanna	Neg	Neg	**–**	**–**	**–**
	JP-5-47	Adult, M	Bradford	Neg	Neg	**–**	**–**	**–**
	JP-5-53	Juvenile, F	Wayne	Pos	Pos	*S. cristata/S. wenzeli*-like	*S. cristata/S. wenzeli*	*S. albifronsi*-like
	JP-5-56	Adult, F	Susquehanna	Pos	Pos	*S. cruzi*	NA	*S.* sp.
	JP-5-58	Adult, F	Susquehanna	Pos	Pos	NA	NA	*S.* sp.
	JP-5-59	Adult, F	Bradford	Pos	Neg	**–**	**–**	**–**
	JP-5-60	Adult, M	Pike	Neg	Neg	**–**	**–**	**–**
	JP-5-61	Adult, M	Pike	Neg	Neg	**–**	**–**	**–**
	JP-5-65	Juvenile, F	Susquehanna	Pos	Neg	**–**	**–**	**–**
	JP-5-66	Juvenile, F	Susquehanna	Neg	Neg	**–**	**–**	**–**
	JP-5-68	Adult, F	Bradford	Pos	Neg	**–**	**–**	**–**
	JP-5-72^*^	Adult, M	Wyoming	Pos	Pos	NA	NA	NA
Gray fox	23,007	Adult, F	Clearfield	Pos	Neg	**–**	**–**	**–**
	23,008	Adult, M	Blair	Pos	Neg	**–**	**–**	**–**
	23,009	Adult, F	Blair	Pos	Neg	**–**	**–**	**–**
	230,012	Adult, F	Centre	Neg	Neg	**–**	**–**	**–**
	230,013	Juvenile, F	Centre	Neg	Neg	NA	NA	NA
	230,014	Juvenile, F	Centre	Neg	Neg	NA	NA	NA
Red fox	RF-PA-23-5	Adult, M	Perry	Neg	Neg	**–**	**–**	**–**
	RF-PA-23-9	Adult, F	Centre	Neg	Neg	NA	NA	NA
	RF-PA-23-10	Adult, M	Elk	Pos	Pos	*S. cristata/S. wenzeli*-like	*S.* sp.	*S. albifronsi*-like
	RF-PA-23-23 (USDA-PA1)	Adult, M	York	Pos	Pos	*S. gjerdei*-like	*S.* sp.	NA
	25-RF1	Adult, F	Centre	Pos	Neg	**–**	**–**	**–**
	26-RF1	Adult, F	Dauphin	Neg	Neg	**–**	**–**	**–**
	26-RF2	Adult, M	Dauphin	Neg	Neg	**–**	**–**	**–**
	26-RF3	Adult, F	Dauphin	Neg	Neg	**–**	**–**	**–**

*Pos* positive, *Neg* negative, *M* male, *F* female

^*^28S rRNA was also successfully amplified in one of the coyotes (#JP-5-72)

### Microscopical examination

For collection of *Sarcocystis* sporocysts, each ileum sample was homogenized in a domestic blender in 100 ml of water. The homogenate was mixed with 100 ml of undiluted Clorox (7.5% sodium hypochlorite) and, after 30 min, centrifuged; sporocysts were collected from the sediment, as described previously [1, 22, 23]. To prepare sporocysts for microscopy, the sediment was washed three times with distilled water by centrifugation (10 min at 800 × g) to remove residual bleach. A small drop of the final suspension was placed onto a glass slide, covered with a coverslip, and examined as a wet mount. Processed samples were stored at – 20 ° C pending DNA extraction.

### DNA extraction and PCR amplification

Genomic DNA was extracted from microscopically positive intestinal homogenates of the foxes and the coyotes containing sporocysts using a the Qiagen DNeasy^®^ PowerSoil^®^ Pro Kit (Hilden, Germany), following the manufacturer’s protocol (sample details in Table 1).

Initial molecular screening was done using Cytochrome c oxidase subunit 1 (*COI*). Next, we attempted to amplify other markers such as *18S* ribosomal RNA (*18S* rRNA), 2*8S* ribosomal RNA (*28S* rRNA) and the Internal Transcribed Spacer 1 (*ITS1*) from the 13 (of 24) samples that initially tested PCR positive. Clean sequences were obtained from 9 of these 13 intestinal homogenates. The remaining four samples produced mixed or poor-quality chromatograms, suggesting contamination or the presence of mixed infections; these four were therefore excluded from further analysis. As shown in Table 1, in the nine evaluable samples, not all markers amplified consistently. This variation may reflect differences in parasite load among samples, partial degradation of intestinal DNA, or mixed *Sarcocystis* infections. To ensure the reliability of our results, we used only sequences that showed clear chromatogram peaks and unambiguous matches in BLAST^n^ analyses.

For the PCR amplification, S3f/Primer BSarc primer pairs [24, 25] were used for partial *18S* rRNA sequence amplification, KL1/KL3 for partial *28S* rDNA fragment gene amplification [26], P-ITS1F/P-ITS1R primers were used to amplify a partial *ITS1* region [26] and partial *COI* sequences were amplified with the help of SF1/SR5 and SF1/SR9 primers [27].

Each PCR reaction was prepared with a total volume of 25 µl, comprising 2 µl of DNA template, 12.5 µl of Platinum Hot Start PCR Master Mix (Invitrogen, Waltham, Massachusetts, USA), 1 µl of each primer at a concentration of 10 pmol/µl (Integrated DNA Technologies, Coralville, Iowa, USA), and 8.5 µl of molecular-grade water. The thermal cycling program included an initial denaturation step at 94 °C for 3 min, followed by 35 cycles of denaturation at 94 °C for 30 s, annealing at 60 °C for 30 s, and extension at 68 °C for 20 s, concluding with a final extension at 68 °C for 5 min. The thermal cycling conditions were same for all the gene markers. No positive controls were used for PCR; water was used as a negative control. The amplicons were sequenced directly using the same forward and reverse primers as for the PCR.

PCR products were separated on a 2% agarose gel, and their sizes estimated by reference to a 100 bp Plus DNA Ladder (Promega, Madison, WI, USA). The amplified products were purified using ExoSAP treatment as described by [28]. These purified products were subjected to bi-directional Sanger sequencing to Psomagen, Inc. (Rockville, MD, USA), utilizing an ABI 3500xl Genetic Analyzer (Applied Biosystems™, Waltham, MA, USA).

### Sequence analysis

Chromatograms were manually inspected. Sequences were edited, quality-trimmed and assembled using the de novo sequence analysis implemented in Geneious Prime® 2025.1.3 (www.geneious.com) and submitted to GenBank (Table 2). The resulting consensus sequences were compared with reference sequences in GenBank using BLAST^n^ (Basic Local Alignment Search Tool; [29] accessed on 04.21.2026). For each gene, only sequences meeting quality thresholds were included in downstream analyses, as multiple amplifications were obtained for each *Sarcocystis* species from the intestinal homogenates.

**Table 2:** Homogeneity table showing interspecific nucleotide sequence similarities between *Sarcocystis* species identified in coyotes and foxes from Pennsylvania, USA, and closely related taxa available in GenBank

Animal	Lab ID	Locus	Fragment length (bp)	BLAST^n^ identity (#)	*Sarcocystis* spp. (GenBank accession number)
Coyote	JP-5–12	*18S* rRNA	432	99.8% *S. cruzi* (MK981204, MN121576), 98.6% *S. levinei* (KU247922, MG957189) and others	*S. cruzi* (PZ435576)
		*COI*	993	89% *S. hjorti* (OM906806, KY973288) and others	*S.* sp. (PZ441411)
	JP-5–17	Mixed	NR	NR	NR
	JP-5–35	*COI*	844	90% *S. hjorti* (OP617391, KY973288) and others	*S.* sp. (PZ441412)
	JP-5–39	*COI*	952	89.5% *S. hjorti* (OM906806, KY973288) and others	*S.* sp. (PZ441413)
	JP-5–53	*18S rRNA*	576	100% *S. cristata* (MT676454) and *S.* sp. (PX571990), 99.8% *S. wenzeli* (MT756990, MT756993) and others	*S. cristata/S. wenzeli*-like (PZ435620)
		*ITS1*	356	99.7% *S. wenzeli* (PQ192598), 91% *S.* sp. (PV364797) and others	*S. cristata/S. wenzeli*-like (PZ437088)
		*COI*	1009	99% *S. albifronsi* (MH138310), 98.5% *S. wenzeli* (MT761700), 98.1% *S. cristata* (MT681118) and others	*S. albifronsi*-like (PZ441414)
	JP-5–56	*18S rRNA*	632	100% *S. cruzi* (LC570810, KC209738, KT901167), 98.6% *S. levinei* (KU247922, KU247916) and others	*S. cruzi* (PZ435655)
		*COI*	988	89.5% *S. hjorti* (OM906806, KY973288) and others	*S.* sp. (PZ441415)
	JP-5–58	*COI*	953	90.2% *S. hjorti* (OM906806, KY973288) and others	*S.* sp. (PZ441416)
	JP-5–72	*28S rRNA*	246	97.6% *S. wenzeli* (MT756986, MT756987), 97.2% *S. cristata* (MT676455), 96.5% *S. albifronsi* (EF079885) and others	*S. cristata/S. wenzeli*-like (PZ437670)
Gray fox	230,013	Mixed	Mixed	NR	NR
	230,014	Mixed	Mixed	NR	NR
Red fox	RF-PA-23–9	Mixed	Mixed	NR	NR
	RF-PA-23–10	*18S rRNA*	551	99.8% *S. cristata* (MT676454)*, S.* sp. (PX571990), 99.6% *S. wenzeli* (MT756990, MT756993) and others	*S. cristata/S. wenzeli*-like (PZ435687)
		*ITS1*	414	79.3% *S. cristata* (MT676453) and others	*S.* sp. (PZ439029)
		*COI*	756	98.8% *S. albifronsi* (MH138310), 98.5% *S. anasi* (MH138311), 97.7% *S. rileyi* (OR423018), *S. wenzeli* (MT761700), *S. cristata* (MT681118) and others	*S. albifronsi*-like (PZ441417)
	RF-PA-23–23	*18S rRNA*	618	99% *S. gjerdei* (LC481030, LC349476,), 98.7% *S. iberica* (KY973316), 98.5% *S. venatoria* (KY973324, KY973325), *S. hjorti* (GQ250990) and others	*S. gjerdei*-like (PZ436061)
		*ITS1*	348	99% *S.* sp. (ON209177), 99% *S. tenella* (MF039318, MF039319) and others	*S.* sp. (PZ437089)

*NR* no results

Prior to phylogenetic reconstruction, all sequences were trimmed at each end so that all sequences were of same length for the inference of accurate and reliable evolutionary relationships. The web server GUIDANCE2 [30] was used to align and remove ambiguously aligned positions. Specifically, the sequences were aligned with the MAFFT algorithm under the options Max-Iterate: 1000 and Pairwise Alignment Method: –localpair. Furthermore, they were also aligned and checked for any discrepancy in Geneious Prime^®^ 2025.1.3 (www.geneious.com) software with MAFFT algorithm under the following parameters: Algorithm: L-INS-i, scoring matrix: 200PAM / k = 2, gap open penalty: 1.53 and offset value: 0.123. Phylogenetic relationships were reconstructed under the maximum likelihood (ML) criterion. ML analyses were performed with the program IQ-TREE version 1.6.12 [31]. The analyses were run with the options –m MFP –b 1000. The model selected based on the BIC criterion for were K2 + I, HKY + G, and HKY + I, for *18S rRNA*, *COI* and *ITS1*, respectively. The final dataset included 23 taxa and 514 positions for *18S* rRNA, 20 taxa and 694 positions for *COI* and 14 taxa and 442 positions for *ITS1* genes. Isolates of *Eimeria tenella* were used to root the trees.

## Results

### Prevalence

**Fig. 1:**
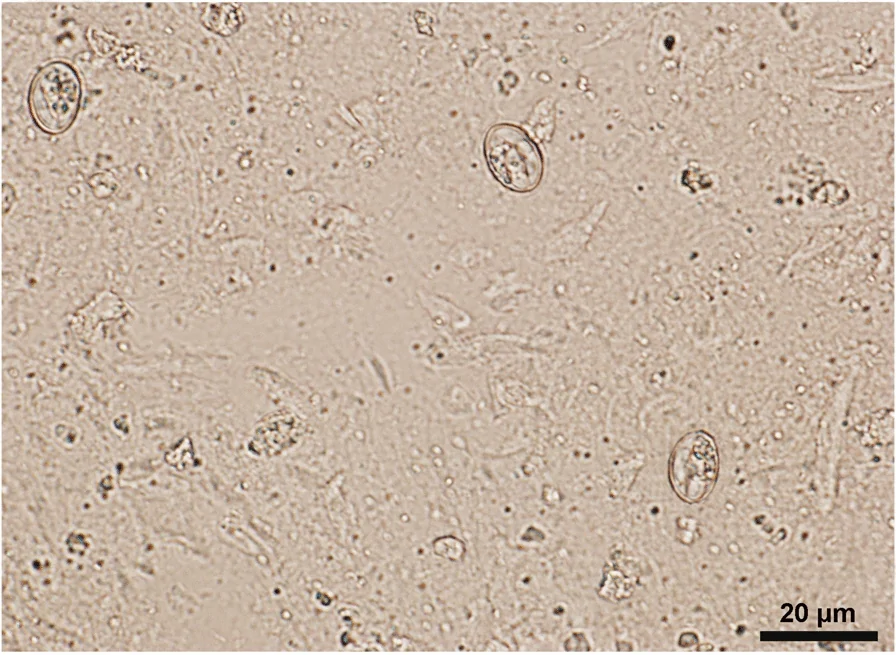
*Sarcocystis* sporocysts in intestinal homogenates of a coyote (#JP-5-53) from Pennsylvania, USA

*Sarcocystis* sporocysts were detected in gray foxes: 3/6 (50%), red foxes: 3/8 (37.5%) and coyotes: 18/26 (69.2%) microscopically (Fig. 1; Table 1).

**Fig. 2:**
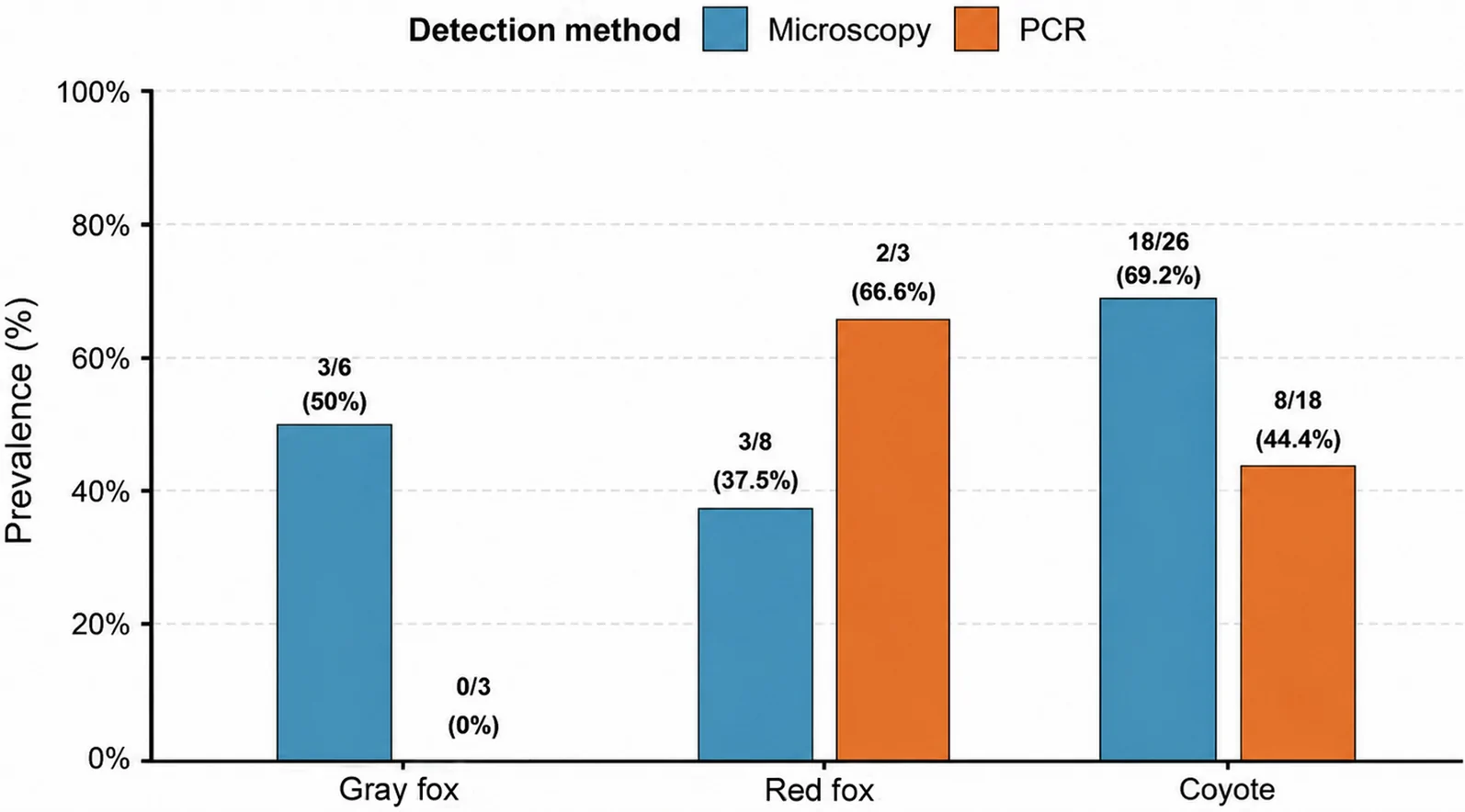
Graph showing the prevalence of *Sarcocystis* sporocysts in the intestines of coyotes and foxes from Pennsylvania, USA

Prevalence estimates with 95% Wilson confidence intervals and graphical comparisons of detection methods are provided in Fig. 2. The PCR successfully amplified most positive samples, however, a few sequences yielded contaminated or poor-quality reads and could not be reliably amplified. This may reflect low parasite DNA concentration, PCR inhibition, DNA degradation, or the presence of mixed infections interfering with amplification efficiency.

### Molecular and phylogenetic analysis

To resolve the identity of *Sarcocystis* species detected in coyotes and foxes under the study, we performed a phylogenetic analysis using three distinct markers: *18S* rRNA, *28S rRNA*, *COI*, and *ITS1*. Sequences derived from the coyotes and foxes clustered within multiple well-supported *Sarcocystis* lineages, indicating the presence of diverse species associated with both ungulate and avian intermediate hosts.: *Sarcocystis cruzi*: The *18S* rRNA sequences (432 bp and 632 bp) showed 99.8–100% identities to reference *S. cruzi* sequences (IH: *Bos taurus*, DH: carnivores), confirming species identity. Closely related sequences included *S. levinei* (98.6% identity; IH: *Bubalus bubalis*), consistent with their known phylogenetic proximity (Table 2).

*Sarcocystis cristata/Sarcocystis wenzeli*-like: *18S* rRNA (576 bp and 551 bp), *28S* rRNA (246 bp) and *ITS1* (356 bp) sequences showed 97.6–100% identity to *S. cristata* and *S. wenzeli* (IH: birds, DH: carnivores). These sequences formed part of a closely related cluster that also included *S. albifronsi* and other undescribed *Sarcocystis* spp., reflecting limited resolution of conserved loci within this group (Table 2).

*Sarcocystis albifronsi*-like: *COI* sequences (1009 bp and 756 bp) showed 98.8–99% identity to *S. albifronsi* (IH: birds, DH: fox and lynx), confirming its presence. These sequences also showed high similarity (98.5%) to *S. anasi*, *S. wenzeli*, and *S. rileyi*, consistent with their close evolutionary relationships (Table 2).

*Sarcocystis gjerdei*-like: *18S* rRNA sequences (618 bp) showed 99% identity to *S. gjerdei* (IH: sika deer), confirming its presence. These sequences also showed high similarity (98.5–98.7%) to *S. iberica*, *S. venatoria*, and *S. hjorti* showing closely proximity between the species (Table 2).

*Sarcocystis* sp.: *COI* sequences (993 bp, 844 bp, 952 bp, 988 bp, 953 bp) showed approx. 79–90% identity to reference taxa such as *S. hjorti* (IH: sika deer, DH: foxes). This level of divergence suggests the presence of potentially undescribed *Sarcocystis* lineages. *ITS1* sequences from these isolates showed variable similarity, with some clustering near *S. tenella* or other ungulate-associated taxa, further supporting genetic diversity within the dataset (Table 2).

The mean intraspecific sequence identity for the *COI* gene was 99.2% among *Sarcocystis* sp. isolates and 98.4% among *S. albifronsi*-like isolates, whereas the mean interspecific identity between these two taxa (i.e., *Sarcocystis* sp. and *S. albifronsi*-like isolates) was 82.3%. For the *18S* rRNA gene, the mean intraspecific identity was 99.8% for *S. cruzi* and 95.1% for *S. cristata/S. wenzeli*-like isolates, while the mean interspecific identity between these taxa and the *S. gjerdei*-like species was 81.8%. For the *ITS1* region, the mean interspecific identity among the sequences under study was comparatively low at 40.4%.

**Fig. 3:**
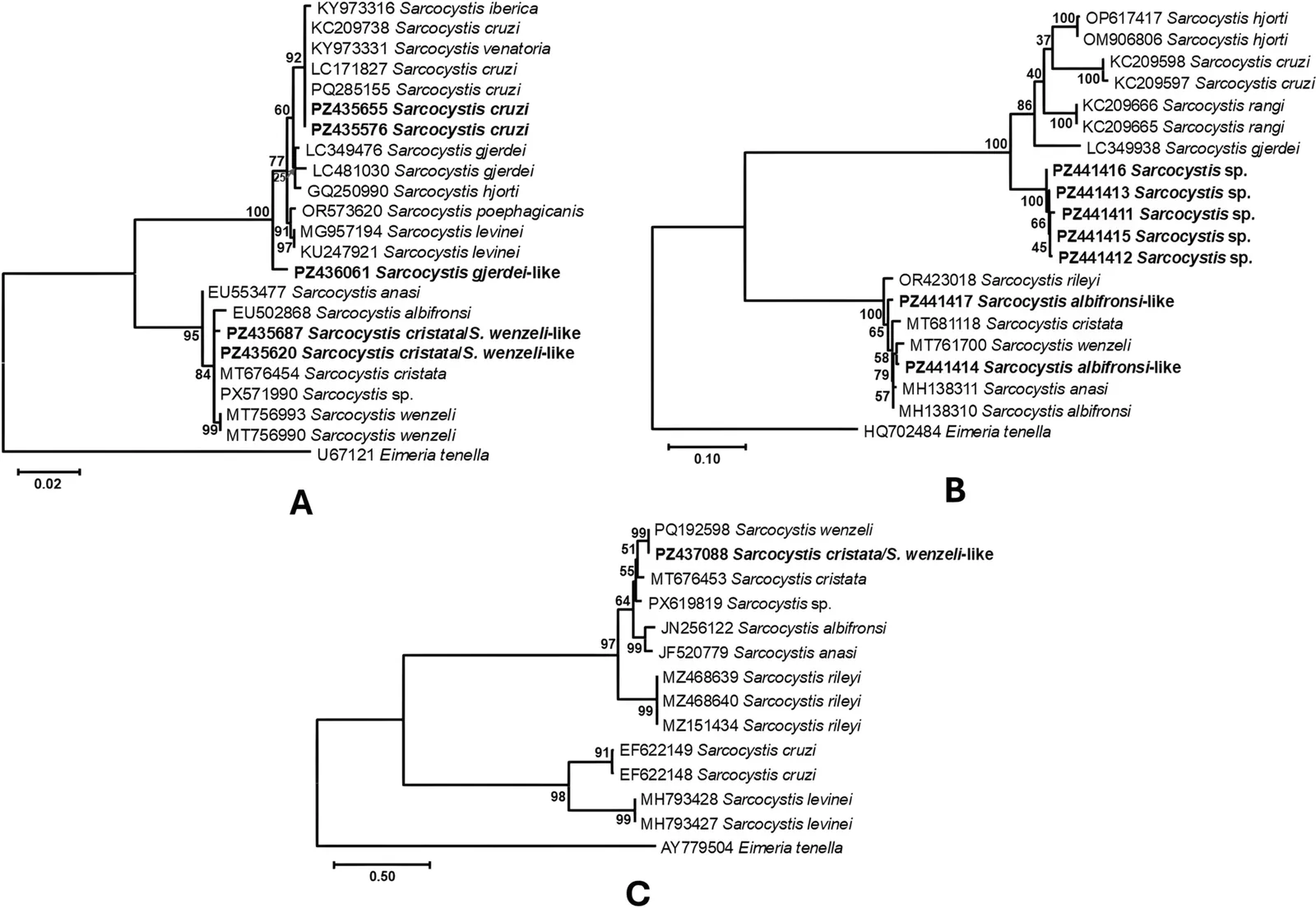
Maximum-likelihood tree based on **A** *18S* rRNA, **B** *COI*, and **C** *ITS1* genetic markers showing the placement of coyotes and foxes derived *Sarcocystis* sequences from Pennsylvania, USA (bold) under study. Reference sequences from known hosts are included for comparison, and isolates of *Eimeria tenella* serves as the outgroup. Scale bars represent the number of substitutions per site

Phylogenetic reconstruction based on the three loci consistently placed all sequences within the genus *Sarcocystis*, while revealing multiple distinct lineages (Fig. 3). In the *18S* rRNA tree, isolates clustered into three main groups: (i) a *S. cruzi* (GenBank: PZ435576, PZ435655), (ii) a *S. wenzeli*/*S. cristata*-like (GenBank: PZ435620, PZ435687), and (iii) a *S. gjerdei*-like (GenBank: PZ436061). These isolates have cattle and birds as the IH origin. These clusters were strongly supported and consistent with the high sequence similarity observed for this conserved marker (Fig. 3A).

The *COI* tree revealed the highest level of divergence among isolates. Several sequences (GenBank: PZ441411, PZ441412, PZ441413, PZ441415, PZ441416) formed strongly supported monophyletic clades with approximately 10% divergence from their closest congeners, suggesting the presence of potentially novel lineages (Fig. 3B).

In contrast, sequences (GenBank: PZ441414) and (GenBank: PZ441417) clustered within a broader clade that includes *Sarcocystis albifronsi*, *S. anasi*, *S. cristata*, and *S. wenzeli*, without strong support for clear separation among these closely related taxa in the *COI* dataset.

This pattern is consistent with the *18S* rRNA phylogeny, which supports a close relationship among these taxa but does not provide sufficient resolution to confidently distinguish between them.

However, the *ITS1* phylogeny offers improved resolution, with sequence (GenBank: PZ437088) clustering more specifically within a *S. wenzeli*-like lineage, suggesting that *ITS1* provides greater discriminatory power for resolving relationships within this group. Other *ITS1* sequences showed lower similarity to reference taxa, indicating additional genetic diversity (Fig. 3C).

Overall, the multilocus phylogeny indicates the presence of multiple, genetically distinct *Sarcocystis* spp. in coyotes and foxes in Pennsylvania, USA.

## Discussion

This study provides multilocus molecular evidence that foxes and coyotes in Pennsylvania harbor diverse *Sarcocystis* spp. confirmed by both microscopy and molecular studies.

Coyotes have long been recognized as competent definitive hosts for *Sarcocystis* spp. [32, 33]. Fecal surveys conducted five decades ago reported variable prevalence ranging between 6 to 88% of *Sarcocystis* sporocysts in wild coyotes in United States populations, although species-level identification was not possible at that time due to reliance on morphology alone. Molecular-based surveys, conducted elsewhere are summarized in Table 3. Our findings extend those observations by providing molecular confirmation of species identity from free-ranging coyotes in the northeastern United States. The high detection rate (60%) observed in this study aligns with prior reports indicating that wild canids (coyotes and foxes) frequently participate in predator–prey transmission cycles involving cervids and livestock (Table 3).

**Table 3:** Molecular studies on *Sarcocystis* sporocysts in feces of wild canids^a^

Host	Country	Region	Year	No. positive/no. tested (%) and other details	*Sarcocystis* species identified	Molecular marker/s employed	Reference
*Canis lupus*	Lithuania	Eastern, southern, and central parts	2021–2025	10/12 (83.3)	*S. arvalis, S. myodes, S. ratti, S.* sp.	*28S* rRNA	[16]
*Canis lupus*	Lithuania	Central and southern parts	2021–2023	12/13 (92.3)	*S. arieticanis, S. alces, S. capracanis, S. capreolicanis, S. gracilis, S. hjorti, S. iberica, S. linearis, S. miescheriana, S. pilosa, S. tenella *and* S. venatoria*	*COI*	[37]
*Canis lupus*	USA	Minnesota	2023	3/3 (100)	*S. cruzi, S. mehlhorni, S. wenzeli*	*18S* rRNA, 2*8S* rRNA, *COI*, *ITS1*	[3]
*Lycalopex gymnocercus*	Argentina	Buenos Aires province	NS	23/131 (17.6)	*S. cruzi, S.* sp.,* S. tenella, S. miescheriana*	*18S* rRNA	[2]
*Nyctereutes procyonoides*	Lithuania	Eastern, southern, and central parts	2021–2025	15/31 (48.4)	*S. arvalis, S. myodes, S. ratti, S.* sp.	*28S* rRNA	[16]
*Nyctereutes procyonoides*	Lithuania	Ukmergė and Tauragė districts	2012	7/13 (53.8)	*S. rileyi*	*ITS1*	[10]
*Nyctereutes procyonoides*	Lithuania	NS	2019–2024	13/26 (50)	*S. alces, S. capracanis, S. capreolicanis, S. gracilis, S. hjorti, S. iberica, S. linearis, S. miescheriana, S. morae, S. tenella, S. venatoria*	*COI*	[36]
*Vulpes vulpes*	Croatia	NS	2021–2024	1/80 (1.3)	*S. arieticanis, S. tenella, S. capracanis, S. cruzi, S. miescheriana, S. capreolicanis, S. gracilis, S. hjorti, S. iberica, S. linearis, S. morae*	*COI*	[15]
*Vulpes vulpes*	Japan	Eastern Hokkaido	2018	1/65 (1.5)	*S. pilosa*	*18S* rRNA, *COI*	[13]
*Vulpes vulpes*	Germany	Brandenburg	2012	19/50 (38), IS	*S. tenella or S. capracanis, S. miescheriana, S. gracilis, S.* spp.,* S. capreolicanis*	*18S* rRNA	[11]
*Vulpes vulpes*	Luthuania	NS	2021–2024	7/42 (16.7)	*S. arieticanis, S. tenella, S. capracanis, S. cruzi, S. miescheriana, S. capreolicanis, S. gracilis, S. hjorti, S. Iberica, S. linearis, S. morae*	*COI*	[15]
*Vulpes vulpes*	Portugal	NS	2021–2024	15/50 (30.0)	*S. arieticanis, S. tenella, S. capracanis, S. cruzi, S. miescheriana, S. capreolicanis, S. gracilis, S. hjorti, S. Iberica, S. linearis, S. morae*	*COI*	[15]
*Vulpes vulpes*	Lithuania	Eastern, southern, and central parts	2021–2025	47/69 (68.1)	*S. arvalis, S. myodes, S. ratti, S.* sp.	*28S* rRNA	[16]
*Vulpes vulpes*	Lithuania	Ukmergė and Panevėžys districts	2012	4/23 (17.4)	*S. rileyi*	*ITS1*	[10]
*Vulpes vulpes*	Czech Republic	9 regions	2019	75/197 (38.1), IS	*S. arieticanis, S. capracanis, S. cruzi, S. miescheriana, S. tenella*	*COI*	[14]
*Vulpes vulpes*	Switzerland	NS	2013–2015	122/126 (96.8) (IS 20)	*S. tenella, S.* sp.*, S. hjorti,* *S. miescheriana, S. gracilis*	*18S* rRNA	[12]

^a^Sporocysts in fecal floats unless stated otherwise

IS = intestinal scrapings

NS = not stated

Red foxes have been molecularly confirmed as definitive hosts for multiple cervid-associated *Sarcocystis* species (Table 3). Molecular surveys using intestinal mucosal scrapings have identified diverse *Sarcocystis* taxa in fox populations [10–16] demonstrating that foxes contribute to environmental dissemination of sporocysts (Table 3). The present findings confirm that red foxes in Pennsylvania similarly harbor many *Sarcocystis* spp. There are no published records of natural transmission of *Sarcocystis* in gray foxes in wild. Transmission studies on gray foxes as DH for *Sarcocystis* species are scarce, and only one study has been performed on gray foxes [34] in which 2 foxes raised in captivity excreted sporocysts and were referred to as *Sarcocystis* sp. The donor host (IH) in both the foxes were white-tailed deer and mule deer. The present study identifies gray foxes (3/6, 50%) as DH for *Sarcocystis* prevalence, albeit molecular analysis produced mixed reads, therefore, the species-level identification could not be performed in them (Table 1). We are also not sure if the Clorox (7.5% sodium hypochlorite) treatment affects the DNA quality of the sporocysts.

Among the detected species, *S. cruzi* warrants special attention due to its well-established canid–bovine life cycle and veterinary and economic importance. Fayer and Johnson [19] demonstrated that dogs fed infected bovine tissues shed *S. cruzi* sporocysts after a short prepatent period. Five decades ago, coyote was proven definitive hosts for *S. cruzi*; calves fed sporocysts from coyote feces developed sarcocysts to maturity and laboratory raised coyotes fed beef from experimentally infected cattle excreted *S. cruzi* sporocysts. Laboratory raised red foxes excreted sporocysts after eating meat from cattle, sheep, and goats experimentally infected with *S. cruzi, S. tenella* or *S. capracanis*, respectively [17]. However, no bioassays have been performed on sporocysts from naturally infected foxes. Previous studies have molecularly identified *S. cruzi* in foxes from Argentina, Czech Republic and Portugal, respectively [2, 14, 15].

Given that coyotes and foxes are closely related to domestic dogs and exhibit similar feeding ecology, they may serve as definitive hosts maintaining transmission in wild and agricultural areas (Table 3).

From an ecological perspective, the diversity of *Sarcocystis* lineages detected in canids likely reflects their role as DH in multiple predator–prey transmission cycles [1]. The detection of *S. cruzi*-like sequences is consistent with well-established cycles involving ungulates as IH [17, 18]. In contrast, the presence of lineages related to *S. wenzeli*, *S. cristata*, and *S. albifronsi*-species, infecting avian IH, suggests additional transmission pathways involving birds [9]. Importantly, although several isolates in this study exhibit substantial genetic divergence (89–90%), particularly at the *COI* locus, we conservatively designated them as *Sarcocystis* spp. rather than assigned to new species. *Sarcocystis wenzeli* is a pathogen of chickens (*Gallus domesticus*) and was recently detected in the intestines of wolves in the USA [3]. Severe encephalitis was reported in free range chickens in Mississippi, USA; schizonts but not sarcocysts were found in lesions [35].

Millions of coyotes and red foxes are present in USA, and they are increasingly adapting to semi-urban lifecycles; thus, they can come in contact with domestic garbage in addition to preying on or eating livestock carcasses. Pennsylvania’s substantial cattle industry, combined with expanding coyote populations and established fox distributions, creates ecological conditions conducive to transmission of these highly enigmatic parasites. Sporocysts shed by wild canids are environmentally resistant and may contaminate feed, pasture, and water sources, facilitating bovine exposure. Pennsylvania supports high densities of white-tailed deer, which serve as intermediate hosts for several *Sarcocystis* species. The detection of multiple *Sarcocystis* species during the study suggests that coyotes and foxes may participate in several parallel predator–prey cycles, potentially involving both wild cervids and domestic cattle, which suggest a need for more comprehensive sampling of isolates and markers.

## Conclusions

This study demonstrates that wild foxes and coyotes in Pennsylvania harbor multiple *Sarcocystis* species, including the cattle-associated parasite *Sarcocystis cruzi*. These findings reinforce the role of coyotes and foxes as definitive hosts contributing to environmental contamination in mixed wildlife–livestock ecosystems. Expanded surveillance integrating molecular tools will be essential to clarify transmission networks and assess implications for livestock health.

## Data Availability

Data supporting the main conclusions of this study are included in the manuscript.

## Abbreviations

DH: Definitive host
IH: Intermediate host
APDL: Animal Parasitic Diseases Laboratory
DNA: Deoxyribonucleic acid
VA: Virginia
USA: United States of America
*COI*: Cytochrome c oxidase subunit 1
*18S*rRNA: *18**S* Ribosomal RNA
*ITS1*: Internal transcribed spacer 1
BLAST: Basic local alignment search tool
PCR: Polymerase chain reaction
WI: Wisconsin
MD: Maryland
MAFFT: Multiple alignment using fast Fourier transform
ML: Maximum likelihood
